# Quality control of Photosystem II: reversible and irreversible protein aggregation decides the fate of Photosystem II under excessive illumination

**DOI:** 10.3389/fpls.2013.00433

**Published:** 2013-10-29

**Authors:** Yasusi Yamamoto, Haruka Hori, Suguru Kai, Tomomi Ishikawa, Atsuki Ohnishi, Nodoka Tsumura, Noriko Morita

**Affiliations:** Graduate School of Natural Science and Technology, Okayama UniversityOkayama, Japan

**Keywords:** Photosystem II, protein aggregation, non-photochemical quenching, photoinhibition, lipid peroxidation, membrane dynamics, thylakoid unstacking, quality control mechanism

## Abstract

In response to excessive light, the thylakoid membranes of higher plant chloroplasts show dynamic changes including the degradation and reassembly of proteins, a change in the distribution of proteins, and large-scale structural changes such as unstacking of the grana. Here, we examined the aggregation of light-harvesting chlorophyll-protein complexes and Photosystem II core subunits of spinach thylakoid membranes under light stress with 77K chlorophyll fluorescence; aggregation of these proteins was found to proceed with increasing light intensity. Measurement of changes in the fluidity of thylakoid membranes with fluorescence polarization of diphenylhexatriene showed that membrane fluidity increased at a light intensity of 500–1,000 μmol photons m^-^^2^ s^-^^1^, and decreased at very high light intensity (1,500 μmol photons m^-^^2^ s^-^^1^). The aggregation of light-harvesting complexes at moderately high light intensity is known to be reversible, while that of Photosystem II core subunits at extremely high light intensity is irreversible. It is likely that the reversibility of protein aggregation is closely related to membrane fluidity: increases in fluidity should stimulate reversible protein aggregation, whereas irreversible protein aggregation might decrease membrane fluidity. When spinach leaves were pre-illuminated with moderately high light intensity, the qE component of non-photochemical quenching and the optimum quantum yield of Photosystem II increased, indicating that Photosystem II/light-harvesting complexes rearranged in the thylakoid membranes to optimize Photosystem II activity. Transmission electron microscopy revealed that the thylakoids underwent partial unstacking under these light stress conditions. Thus, protein aggregation is involved in thylakoid dynamics and regulates photochemical reactions, thereby deciding the fate of Photosystem II.

## INTRODUCTION

Photosynthesis has a well-known light intensity vs. activity profile (Taiz and Zeiger, 2006). Under low light, the rate of photosynthesis increases in proportion to light intensity, and the excitation energy captured by light-harvesting antennas is efficiently transferred to the reaction centers of Photosystems I (PSI) and II (PSII). As the light intensity increases, the rate of photosynthesis gradually decreases and reaches a plateau where no further increase in photosynthesis is seen. Although PSII can be photodamaged at this stage it is quickly repaired, so that the damage and repair of PSII are balanced. With increasing light intensity, PSII avoids stress by dissipating the excessive light energy as heat.

Energy-dependent quenching (qE; Birantais et al., 1980) is a major component of non-photochemical quenching (NPQ) of chlorophyll fluorescence (Genty et al., 1989), and is activated by acidification of the thylakoid lumen attained through H^+^ uptake into the lumen coupled with electron transport (Birantais et al., 1980). This luminal acidification activates violaxanthin de-epoxidase (Yamamoto and Kamite, 1972), which causes de-epoxidation of the xanthophyll cycle carotenoid violaxanthin (Vio) to zeaxanthin (Zea) in light-harvesting complex (LHC) II (Demmig-Adams, 1990). The PsbS protein (Li et al., 2000), a transmembrane protein, is activated by the acidity of the thylakoid lumen and induces conformational changes and reversible aggregation of LHCII (Horton et al., 1996), although its exact location and function are not revealed yet. Thus, the aggregates of LHCII that are generated and stabilized by Zea are crucial for quenching excess energy and avoiding the risk of PSII over-excitation.

When the light intensity increases further, the maximum quantum efficiency of photosynthesis, as measured by chlorophyll fluorescence Fv/Fm, declines significantly. At this stage, photoinhibition prevails over protection and repair, and PSII is in a typical photodamaging state where degradation of the damaged D1 protein takes place (Barber and Andersson, 1992; Aro et al., 1993). Irreversible aggregation or cross-linking of the photodamaged reaction center-binding D1 protein also occurs (Mori and Yamamoto, 1992; Mori et al., 1995; Yamamoto and Akasaka, 1995; Ishikawa et al., 1999; Yamamoto, 2001; Yamamoto et al., 2008; Khatoon et al., 2009; Chan et al., 2012). The irreversible aggregation of the D1 protein is ascribed to the covalent cross-linking of the protein with nearby polypeptides after photooxidative damage to the D1 protein under light stress. Thus far, three cross-linked products of the D1 protein have been identified: the cross-linked products between D1 and D2 (Yamamoto et al., 2008), those between D1 and the α-subunit of cytochrome *b*_559 _(Barbato et al., 1995), and those between D1 and the core antenna chlorophyll binding protein CP43 (Yamamoto and Akasaka, 1995; Yamamoto et al., 2008). These cross-linked products are formed through photoinhibition of PSII via both donor-side and acceptor-side photoinhibition mechanisms, and are seen both *in vitro* and *in vivo* (Ohira et al., 2005). Once the cross-linked products are formed in the thylakoids, it is difficult to remove them by proteases, although some stromal protease(s) are able to degrade them (Ishikawa et al., 1999; Ferjani et al., 2001). Similar cross-linked products of the D1 protein were also observed in cyanobacterial thylakoids exposed to high light intensity (Lupinkova and Komenda, 2004). Thus, it is likely that cross-linking of the D1 protein with neighboring proteins is a general occurrence in oxygenic photosynthetic organisms subjected to excessive light.

Here, we carried out excessive illumination of spinach leaves or thylakoids and measured protein aggregation with 77K chlorophyll fluorescence, the qE of NPQ with pulse-amplitude-modulation (PAM) chlorophyll fluorometry, and membrane fluidity by fluorescence polarization of diphenylhexatriene (DPH). Samples were illuminated at various light intensities to more comprehensively understand the physiological meanings of protein aggregation under light stress. We also observed structural changes of thylakoids under light stress by TEM. These experiments enabled us to understand the nature of dynamic changes in the proteins, lipids, and thylakoid membranes under excessive illumination.

## MATERIALS AND METHODS

### SAMPLE PREPARATION AND PRE-ILLUMINATION CONDITIONS

Fresh spinach leaves were purchased from a local market in Okayama, Japan, and kept at 4°C in the dark room with a sufficient water supply to the roots before use. Intact thylakoid membranes were prepared as previously described (Yamamoto et al., 2004), with the omission of Na-ascorbate from the grinding medium to avoid its effects on the observation of protein photodamage. The thylakoid membranes were suspended in a buffer solution containing 0.1 M sorbitol, 15 mM NaCl, 5 mM MgCl_2_, 30% (v/v) ethylene glycol, and 50 mM Tricine-KOH pH 7.5 (solution A), frozen in liquid nitrogen and stored at -80°C until use. For all experiments, samples were washed once and suspended in solution A without ethylene glycol (solution B). All procedures were carried out in darkness under a green safe light. Chlorophyll concentrations were determined using an 80% acetone extract and a U-3900 spectrophotometer (Hitachi, Tokyo, Japan).

Pre-illumination of spinach leaves was performed with white light from a tungsten-halogen light source (LA-180Me-R, Hayashi, Japan), which was filtered through a flat glass bottle containing CuSO_4_ solution to absorb heat from the light source. Light intensity was measured with a LI-189 photometer (LI-COR, Lincoln, NE, USA). For pre-illumination of the thylakoids, samples were adjusted to 0.1 mg chlorophyll mL^-1^, placed in 0.5 mL transparent plastic tubes and incubated under illumination with white light and various light intensities in a thermostatic water bath at 20°C. The pre-illumination was terminated by transferring the plastic tubes to an ice bath in the dark.

### MEASUREMENT OF CHLOROPHYLL PROTEIN AGGREGATION

Aggregation of chlorophyll-binding proteins was examined by measuring chlorophyll fluorescence emission spectra at 77K. Spinach thylakoids were suspended in solution B at chlorophyll concentrations of 0.01 mg chlorophyll mL^-1^ and frozen in liquid nitrogen. Fluorescence was measured with a fluorescence spectrophotometer (Jasco FP-8300, Japan) equipped with a low temperature unit. The excitation wavelength was 435 nm with a 20 nm band width, while the emission wavelength was 650–750 nm with a 2.5 nm band width. Curve fitting analysis was carried out with JASCO software Spectra Manager attached to the instrument. The six main components were identified according to Stoitchkova et al. (2006), and are referred to as F680 (peak, 681 nm; half band width, 10.1 nm), F685 (peak, 685 nm; half band width, 9.3 nm), F695 (peak, 693 nm; half band width, 9.2 nm), F700 (peak, 700 nm; half band width, 15.8 nm), F720 (peak 720 nm; half band width, 21.9 nm), and F735 (peak 735 nm; half band width 23.4 nm). They correspond to the fluorescence maxima of the trimeric and monomeric forms of LHCII, the PSII reaction center complex, the core antenna complex of PSII, the aggregated trimers of LHCII, the core complexes of PSI, and LHCI, respectively.

### ANALYSIS of PSII ACTIVITY AND qE OF NPQ

Chlorophyll fluorescence parameters Fv/Fm, NPQ, and qE were measured with a Mini-PAM chlorophyll fluorometer (Walz, Effeltrich, Germany). Prior to the measurements, spinach leaves were kept at 4°C in the dark for 2 h. For the Fv/Fm measurement, leaves were illuminated with white light of given light intensities for 1 h at various temperatures from 4 to 40°C (the light samples), and were then incubated in the dark for 10 min at 20°C before measurement. The dark control samples were incubated for 1 h in the dark. For the measurements of NPQ and qE, leaves were illuminated at given light intensities at 20°C for 20 min (the light sample), while the dark samples were incubated in the dark at 20°C for 20 min. The leaves were then incubated in the dark for 5 min and subjected to fluorescence measurement.

### MEASUREMENT OF MEMBRANE FLUIDITY

Three micromolar DPH was added to the thylakoid suspension containing 0.01 mg chlorophyll mL^-1^, and the mixture was incubated for 5 min at 20°C in the dark. Fluorescence polarization was measured with a JASCO fluorescence spectrophotometer (FP-8300) equipped with an automatic fluorescence polarization measuring unit (FDP-837) and a temperature controller (EHC-813). The excitation and emission wavelengths were 360 and 430 nm, respectively, and the band widths of excitation and emission were 5 nm and 20 nm, respectively. The temperature was increased from 5 to 40°C at a rate of 5°C min^-1^.

### ELECTRON MICROSCOPY

The spinach leaves were kept in the dark overnight at 15°C and used for the dark control samples. The light stressed samples were obtained from leaves illuminated for 1 h with white light (light intensity: 1,500 μmol photons m^-2^ s^-1^) after adaptation to the dark. The dark and light-treated leaves were cut into 1 × 3 mm fragments, pre-fixed in 3% paraformaldehyde and 2.5% glutaraldehyde, and post-fixed with 1% OsO_4_. After embedding in plain resin, the samples were thin-sectioned into 70 nm-thick samples with an ultramicrotome (Leica EM UC7, Germany). Electron micrographs were obtained with a transmission electron microscope (Hitachi H-7650).

## RESULTS

### MEASUREMENT OF PROTEIN AGGREGATION BY 77K CHLOROPHYLL FLUORESCENCE

**FIGURE 1:**
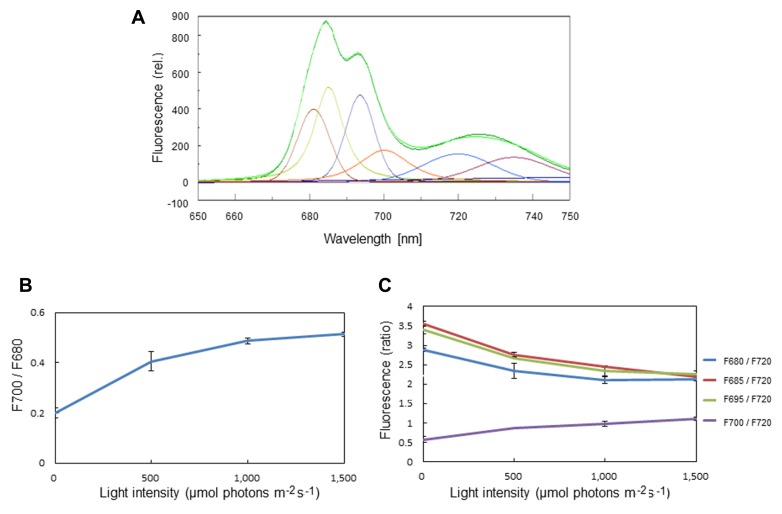
**Chlorophyll fluorescence emission spectra of spinach thylakoids at 77K.** **(A)** A typical fluorescence emission spectrum with Gaussian decompositions from thylakoids kept in the dark. The six main components were identified as F680 (peak, 681 nm), F685 (peak, 685 nm), F695 (peak, 693 nm), F700 (peak, 700 nm), F720 (peak 720 nm), and F735 (peak 735 nm). They correspond to the fluorescence maxima of the trimeric and monomeric forms of LHCII, the PSII reaction center complex, the core antenna complex of PSII, the aggregated trimers of LHCII, the core complex of PSI, and LHCI, respectively. **(B)** The ratio of F700:F680 at various light intensities. The increase in the ratio under excessive light indicates the conversion of free LHCIIs to aggregated ones. The data are the means of three independent measurements ± S.D. **(C)** The ratios of F680:F720 (blue), F685:F720 (red), F695:F720 (green), and F700:F720 (purple) at various light intensities. The light-induced decrease in F680:F720 and increase in F700:F720 indicate LHCII aggregation, while decrease in F685:F720 and F695:F720 suggests aggregation between the D1 protein and CP43. We assumed that the level of F720 is constant at various light intensities. The data are the means of three independent measurements ± S.D.

The formation of LHCII aggregates under excessive illumination has previously been monitored by 77K chlorophyll fluorescence emission spectra (Ruban and Horton, 1992; Stoitchkova et al., 2006; Haferkamp et al., 2010). We carried out the same measurement of 77K chlorophyll emission spectra using spinach thylakoids pre-illuminated with excessive light of various intensities. Through a curve fitting analysis, we determined the amplitude of simple Gaussian curves representing fluorescence emission peaks of the aggregated trimers of LHCII at 700 nm (F700), and free trimeric and monomeric forms of LHCII at 680 nm (F680; **Figure 1A**). LHCII aggregation brought about an increase in the ratio of F700:F680 at 77K (**Figure 1B**). We also determined the ratios of F680, F685, F695, and F700 to F720 (**Figure 1C**), assuming that the level of F720 representing fluorescence from the core complex of PSI is constant over the light intensities examined. The increase in the ratio of F700:F680 as well as that of F700:F690 under excessive light conditions indicates an increase in LHCII aggregation, while the decrease in F680:F690, F685:F690, and F695:F690 suggests a decrease in the level of chlorophyll fluorescence caused by aggregation of related proteins.

### MEASUREMENT OF THYLAKOID MEMBRANE FLUIDITY

**FIGURE 2:**
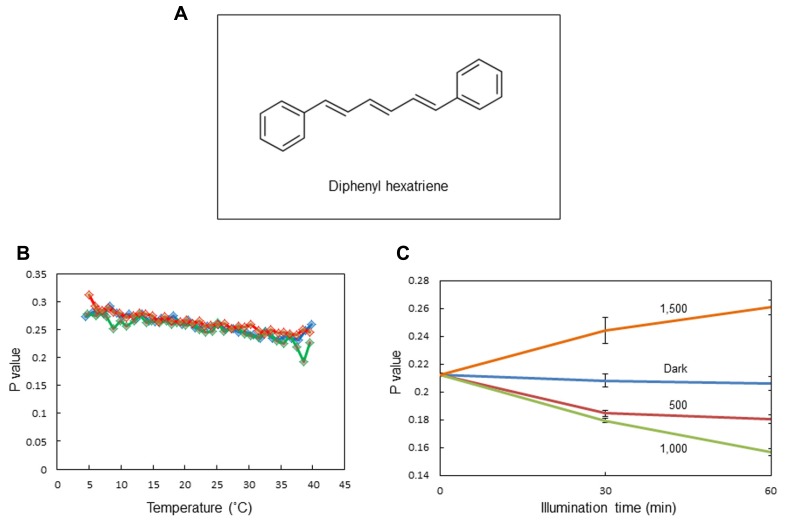
**Assay of the membrane fluidity of spinach thylakoids with fluorescence polarization of diphenylhexatriene (DPH).** **(A)** The chemical structure of DPH. **(B)** The *p*-values of DPH fluorescence polarization in thylakoids incubated at different temperatures from 5 to 40°C. Three different colors indicate three independent measurements. The increase and decrease in the *p*-value indicate a decrease and increase in membrane fluidity, respectively. **(C)** The *p*-values of DPH fluorescence polarization in thylakoids illuminated with white light of different intensities at 20°C. The data are the means of three independent measurements ± S.D.

We observed changes in the membrane fluidity of spinach thylakoids caused by illumination using DPH fluorescence polarization measurements. DPH is a popular fluorescence probe that associates with the hydrophobic region of membranes (**Figure 2A**). A detailed theoretical background of DPH usage has been given previously (Lentz, 1989; Franova et al., 2010). It is widely used to monitor membrane fluidity and the ordering of lipid acyl chains. We first measured changes in membrane fluidity according to the incubation temperature of the thylakoids (**Figure 2B**). As expected, decreases in *p*-values reflecting increases in membrane fluidity were observed when the incubation temperature was increased from 5 to 40°C. Next, we examined the effects of excessive light on the *p*-value (**Figure 2C**). We detected a decrease in *p*-value, representing an increase in membrane fluidity at moderately high light conditions (light intensity: 500–1,000 μmol photons m^-2^ s^-1^), while the *p*-value increased at extremely high light conditions (light intensity: 1,500 μmol photons m^-2^ s^-1^) reflecting a decrease in membrane fluidity.

### MEASUREMENTS OF Fv/Fm, NPQ, AND qE

**FIGURE 3:**
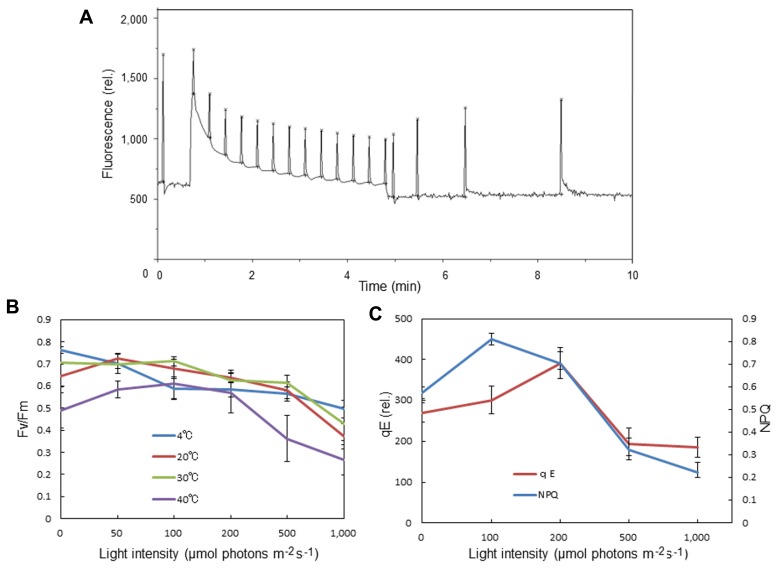
**Effects of pre-illumination of spinach leaves on chlorophyll fluorescence parameters Fv/Fm, NPQ, and qE.** These parameters were measured with a Mini-PAM chlorophyll fluorometer (Walz, Germany). **(A)** A typical time course of fluorescence measurement for NPQ and qE. The light intensity of the continuous illumination was 600 μmol photons m^-2^ s^-1^, and during the illumination several saturating flashes were given to estimate the formation of qE. After continuous illumination, another series of saturating flashes was given to the sample and the fluorescence intensity induced by the flash at the 4 min point in the dark period was used to calculate qE. **(B)** The effects of pre-illumination of spinach leaves at various temperatures on Fv/Fm. The data are the means of three independent measurements ± S.D. **(C)** The effects of pre-illumination of spinach leaves at 20 °C on NPQ and qE. The data are the means of three independent measurements ± S.D.

We observed an increase in the optimum quantum yield of PSII, as measured by chlorophyll fluorescence Fv/Fm, in the spinach leaves that were pre-illuminated with moderately high light for a short period (**Figures 3A, B**). This was not seen at 4°C. Pre-illumination at 20°C also induced an increase in qE in preilluminated leaves compared with dark-adapted leaves (**Figures 3A,C**). These data are consistent with the view that moderate light stress causes mobilization of PSII and LHCII complexes in the grana, thereby maintaining or increasing the efficiency of the PSII reaction. By contrast, prolonged illumination with higher light intensities decreased Fv/Fm as a result of photoinhibition.

A decrease in the chlorophyll fluorescence Fv/Fm always accompanies the photoinhibition of PSII. The decrease in the chlorophyll fluorescence yield may partially be caused by aggregation of the reaction-center binding proteins and the core antenna chlorophyll binding proteins induced by excessive light. In the curve analysis of 77K chlorophyll fluorescence emission spectra, we observed a decrease in fluorescence components emitting at 685 nm (F685) and 695 nm (F695), which is attributable to the fluorescence from the reaction center complex and the core antenna complexes of PSII, respectively (Stoitchkova et al., 2006; **Figure 1**). The aggregation of D1 and CP43, demonstrated previously by western blot analysis to occur under severe light stress (Yamamoto et al., 2008), may be involved in the decrease in F685 and F695.

### LARGE-SCALE STRUCTURAL CHANGES OF THYLAKOIDS UNDER LIGHT STRESS

**FIGURE 4:**
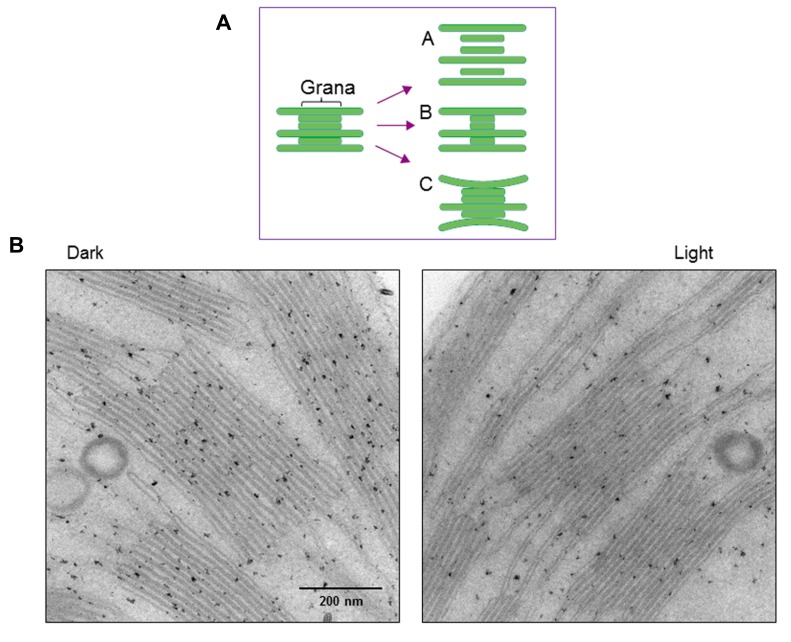
**Changes in thylakoid stacking in the chloroplasts after strong illumination of spinach leaves.** **(A)** A diagram showing possible structural changes in the grana after illumination. Models A, B and C correspond to light-induced unstacking of the grana, shrinkage of the grana and bending of the stromal thylakoids outward. **(B)** Electron micrographs obtained by TEM. Model C in **(A)**, which shows partial thylakoid unstacking, fits best with the present results.

Structural changes of thylakoids occur not only at a molecular level but also on a larger scale. We previously observed strong light-induced unstacking of the grana* in vitro* using isolated spinach thylakoids (Khatoon et al., 2009), where membrane stacking and unstacking were estimated by measuring the chlorophyll content in heavy fractions representing the grana, after fractionation of the grana and stroma thylakoids by digitonin treatment and low speed centrifugation (Chow et al., 1980). Digitonin is a non-ionic detergent effective in separating the grana from the stroma thylakoids. Using this method, we observed that strong illumination induces a decrease in the grana fraction, so concluded that the grana show unstacking under light stress (**Figure 4A**). However, there may be other possible explanations. Light-induced shrinkage of the grana should also decrease the amount of pellet that results from centrifugation of digitonin-treated thylakoids. Light-induced bending of the stromal thylakoids outward, corresponding to a partial unstacking of the thylakoids, may result in an increase in grana margins and a decrease in the grana core.

To more directly observe structural changes in the grana under light stress, we examined spinach thylakoid membranes using TEM (**Figure 4B**). As the chemical fixation of isolated spinach thylakoids may cause various artifacts, we used spinach leaves instead. Outward-bending of the stromal thylakoids was observed after strong illumination (light intensity: 1,500 μmol photons m^-2^ s^-1^) of the spinach leaves for 1 h. Accordingly, the size of the grana core appeared to be reduced after strong illumination.

## DISCUSSION

### PROTEIN AGGREGATION

Among the components that emit chlorophyll fluorescence at 77K, F700 was shown to be directly related to LHCII aggregation. The behavior of F700 in response to the acidic luminal pH and addition of antimycin A, which inhibits qE, showed that qE is stimulated or inhibited by the enhancement or disruption of LHCII aggregation, respectively (Horton et al., 1991). We detected an increase in F700 under illumination of the thylakoids with excessive light over a wide range of light intensities from 500 to 1,500 μmol photons m^-2^ s^-1^ (**Figure 1**). It is interesting to note that F680, 685, and 695 decreased with increased light intensity, confirming that the reaction center binding D1 protein and core antenna chlorophyll binding protein CP43 form irreversible aggregates under strong illumination (Yamamoto, 2001; Yamamoto et al., 2008).

In the measurement of reversible aggregation of LHCII by linear and circular dichroism spectroscopy, changes in the conformation of chlorophylls *a* and *b*, and xanthophyll molecules were suggested (Ruban et al., 1997). It is likely that the Zea molecules in aggregated LHCII adopt a particular orientation suitable for the dissipation of excessive excitation. However, we do not know whether this stabilizes the aggregated LHCII or directly participates in the quenching process. It was suggested that Zea directly quenches the excessive singlet excitation energy of chlorophyll *a* (Frank et al., 2000), while the results obtained by an *in vitro* study suggest that qE is generated when Zea induces LHCII aggregation (Gruszecki et al., 2006). Recent reconstitution experiments with liposomes containing LHCII, Zea, and PsbS support a positive role for all these components in qE (Wilk et al., 2013). Although LHCP aggregation in the qE mechanism is well documented through extensive studies, the exact physiological meanings of LHCII aggregation are still not completely clear (Duffy et al., 2013). By contrast, the mechanism of formation and physiological meanings of irreversible protein aggregation under severe light stress have been more clearly elucidated (Yamamoto, 2001; Yamamoto et al., 2008).

### MOLECULAR CROWDING IN THE THYLAKOID MEMBRANES

The thylakoid membranes are crowded with proteins and protein complexes, such that lateral diffusion of these components is considerably restricted (Kirchhoff et al., 2008a, 2008b). The crowded conditions are most typically seen in the grana regions of higher plant chloroplasts where the PSII complexes are enriched and sometimes show highly packed semi-crystalline arrays (Johnson et al., 2011). The thylakoids have to respond to incident light to efficiently capture light energy when the light is relatively weak and behave appropriately when the light is excessive. Thus, a suitable “crowd control mechanism” must work to optimize the photochemical reactions. The most important process in this is likely to be regulation of the lateral diffusion of PSII/LHCII supercomplexes and other related components on the thylakoids under fluctuating light conditions in the natural environment. In the grana thylakoids, overcrowded PSII/LHCII complexes may cause unnecessary overlap or aggregation of antenna complexes, thereby decreasing the efficiency of excitation energy trapping. Overcrowding also prevents the smooth movement of protein molecules on the membranes. By contrast, dilution of the PSII/LHCII supercomplexes in the thylakoids was shown to induce partial dissociation of minor LHCII complexes from the supercomplexes, leading to inefficient excitation trapping and transfer (Haferkamp et al., 2010). These data demonstrated that a suitable packing density of the PSII, as is realized in the natural grana, is important for efficient energy transfer from the antenna to the reaction center.

### MOBILE LIPIDS IN THE THYLAKOIDS AND CHANGES IN THE ORGANIZATION OF PHOTOSYSTEM II AND LHCII UPON EXCESSIVE ILLUMINATION

Lipids are known to change the degree of saturation of constituent fatty acids, depending on the ambient temperature. This enables intrinsic proteins and lipid-soluble plastoquinones in the thylakoids to move over a wide range of temperatures. Fatty acid desaturases change saturated fatty acids to unsaturated ones when plants are exposed to lower temperatures, while saturases, which catalyze the reverse reaction, have not yet been identified. Polyunsaturated fatty acids become a target for lipid peroxidation, which may cause damage to proteins and induce irreversible aggregation or the cross-linking of proteins. Cross-linking is also expected with thylakoid lipids, which may affect the fluidity of thylakoid membranes, and therefore mobility of the membrane components.

As membrane fluidity determines the lateral diffusion of lipids and proteins on the thylakoid membranes, the measurement of fluidity is an important but difficult task. Thus far, three methods have been successfully employed for the study of thylakoid membrane fluidity: fluorescence recovery after photobleaching (FRAP; Sarcina et al., 2003, 2006; Goral et al., 2010, 2011; Johnson et al., 2011; Herbstova et al., 2012; Kirchhoff et al., 2013), electron paramagnetic resonance (Tardy and Havaux, 1997; Kota et al., 2002), and fluorescence polarization measurement with a suitable fluorescence probe such as DPH (Yamamoto et al., 1981; Ford and Barber, 1983). Following FRAP measurements, cyanobacterial thylakoids and chloroplast grana thylakoids were shown to be relatively immobile, being limited in the lateral movement of supermolecular complexes (Sarcina et al., 2003; Herbstova et al., 2012). In particular the grana are highly crowded with PSII/LHCII complexes and it is apparently not easy for the PSII/LHCII supermolecular complexes to move around freely in these areas (Kirchhoff et al., 2008a). Indeed, only a small fraction of the membrane areas in the grana is mobile and the diffusion constants of the molecules in the mobile areas were experimentally determined to be small. Importantly, strong illumination increased membrane fluidity in cyanobacterial thylakoids (Sarcina et al., 2006) and in the grana of higher plant chloroplasts (Herbstova et al., 2012), thereby inducing mobilization of the PSII complexes.

These and other related studies have opened a new research area investigating light stress in terms of changes in the molecular arrangement of thylakoids, where the roles of LHCII aggregation in the qE process of NPQ and D1 protein and CP43 aggregation in photoinhibition are reevaluated. It should be noted here that all the methods described above to measure membrane fluidity are useful to monitor overall fluidity change in thylakoid membranes. However, they are not suitable to monitor specific changes in local membrane fluidity, in particular around the PSII/LHCII supercomplexes, under light stress. In spite of these difficulties, we monitored changes in thylakoid fluidity with the fluorescence polarization of DPH. We detected an increase in membrane fluidity under moderate light stress and a decrease under severe light stress (**Figure 2**).

The molecular mechanisms of increased membrane fluidity under moderate light stress are not fully understood, but there are several possibilities. Protein phosphorylation is known to be a driving force for the lateral movement of LHCII in thylakoid membranes (Allen, 1992, 2003). Under moderately high light conditions, light-induced phosphorylation of proteins takes place, which drives the lateral diffusion of PSII and LHCII complexes in the grana. This idea is supported by the study of thylakoid kinase mutants by Bonardi et al. (2005), in which no structural alteration occurred in the thylakoid kinase mutants *stn8* or the double mutant *stn7/8* of *Arabidopsis thaliana* (Herbstova et al., 2012). Many proteins are phosphorylated in the thylakoid membranes depending on different environmental conditions (Vener, 2007). Previously, degradation and aggregation of the D1 protein in spinach grana thylakoids under moderate heat stress were shown to be affected by protein phosphorylation and dephosphorylation (Komayama et al., 2007). Subsequently, this was studied more extensively using *stn7*, *stn8*, and *stn7/8* *A. thaliana* mutants under high light stress, when it was demonstrated that not only degradation and aggregation of the D1 protein but also the overall structure of the thylakoid membranes is regulated by the reversible phosphorylation of thylakoid proteins (Fristedt et al., 2009). Thus, it is likely that protein phosphorylation plays an important role in the dynamics of thylakoids in higher plant chloroplasts. Indeed, mobilization of thylakoid proteins under moderate light stress increased qE of NPQ and even transiently activated PSII in the present study (**Figure 3**).

### STRUCTURAL CHANGES OF THYLAKOIDS

Structural changes of the thylakoids under light stress are important in the quality control of PSII. We observed outward bending of the thylakoids at the margins of the grana under relatively strong illumination (**Figure 4**). The mechanism of the partial unstacking of the thylakoids remains to be determined, although, as described above, protein phosphorylation and dephosphorylation may be involved in this process. Recently, CURVATURE THYLAKOID 1 (CURT1) proteins responsible for the induction of membrane curvature were identified in *A. thaliana* (Armbruster et al., 2013), and it would be interesting to determine the effect of light stress on their function. These proteins are enriched at grana margins and are suggested to modify the thylakoid architecture, including grana stacking. Rearrangement of protein complexes in the grana may play crucial roles in grana stacking/unstacking.

We postulate that the outward bending and partial unstacking of the thylakoids can be physiologically explained as follows. First, the outward bending and partial unstacking of the grana would increase the area of the grana margins, which would aid degradation of photo-damaged D1 protein by specific protease(s). FtsH proteases are one candidate for the removal of photodamaged and heat-damaged D1 protein in the thylakoid (Kamata et al., 2005; Komenda et al., 2006; Yoshioka et al., 2006), and the grana margins are the sites of FtsH protease assembly prior to hexameric protease activation and reaction with damaged D1 protein in PSII that has moved from the grana core (Yoshioka et al., 2010). Increase in the area of the grana margins is necessary for the swift repair of damaged D1 proteins. Second, partial unstacking may prevent the production of ROS in the grana by stimulating the free movement of PSII and LHCII complexes. The mobilization of PSII and LHCII complexes should prevent the irreversible aggregation of the complexes and production of ROS from the impaired PSII in the aggregates. Indeed, previous work showed that thylakoid stacking artificially induced by the addition of MgCl_2_ stimulates production of hydroxyl radicals under light stress (Khatoon et al., 2009). Thus, the partial membrane unstacking observed under light stress appears to be a dynamic way of controlling the quality of PSII.

## CONCLUSION

Reversible and irreversible aggregation of LHCII and PSII complexes under moderate and strong light stresses appear to be closely related to the qE component of NPQ and the photoinhibition of PSII, respectively. Although protein aggregation is a subtle phenomenon observed under light stress, it plays a crucial role in the quality control of PSII. It may also be related to the microscopic rearrangement of PSII/LHCII super complexes in the thylakoids and to thylakoid unstacking under light stress, although further studies are required to fully understand the details of these processes.

## AUTHOR CONTRIBUTIONS

Yasusi Yamamoto organized and wrote the manuscript. Haruka Hori, Suguru Kai, Tomomi Ishikawa, Atsuki Ohnishi, and Nodoka Tsumura carried out the experiments and obtained the data presented here. Noriko Morita organized the data and prepared the figures.

## Conflict of Interest Statement

The authors declare that the research was conducted in the absence of any commercial or financial relationships that could be construed as a potential conflict of interest.

## References

[B1] Allen J. F. (1992). How does protein phosphorylation regulate photosynthesis? *Trends Biochem. Sci.* 17 12–17 10.1016/0968-0004(92)90418-9 1585448

[B2] Allen J. F. (2003). State transitions – a question of balance. *Science* 299 1530–1532 10.1126/science.1082833 12624254

[B3] Armbruster U. Labs M. Pribil M. Viola S. Xu W. Scharfenberg M. (2013). *Arabidopsis* CURVATURE THYLAKOID1 proteins modify thylakoid architecture by inducing membrane curvature. *Plant Cell* 25 2661–2678 10.1105/tpc.113.113118 23839788 PMC3753390

[B4] Aro E. M. Virgin I. Andersson B. (1993). Photoinhibition of Photosystem II. Inactivation, protein damage and turnover. *Biochim. Biophys. Acta* 1143 113–134 10.1016/0005-2728(93)90134-2 8318516

[B5] Barbato R. Friso G. Ponticos M. Barber J. (1995). Characterization of the light-induced cross-linking of the alpha-subunit of cytochrome b559 and the D1 protein in isolated photosystem II reaction centers. *J. Biol. Chem.* 270 24032–24037 10.1074/jbc.270.41.24032 7592601

[B6] Barber J. Andersson B. (1992). Too much of a good thing: light can be bad for photosynthesis. *Trends Biochem. Sci.* 17 61–66 10.1016/0968-0004(92)90503-2 1566330

[B7] Birantais J.-M. Vernotte C. Picaud M. Krause G. H. (1980). Chlorophyll fluorescence as a probe for the determination of the photo-induced proton gradient in isolated chloroplasts. *Biochim. Biophys. Acta* 591 198–202 10.1016/0005-2728(80)90233-9 6155943

[B8] Bonardi V. Pesaresi P. Becker T. Schleiff E. Wagner R. Pfannschmidt T. (2005). Photosystem II core phosphorylation and photosynthetic acclimation require two different protein kinases. *Nature* 437 1179–1182 10.1038/nature04016 16237446

[B9] Chan T. Shimizu Y. Pospisil P. Nijo N. Fujiwara A. Taninaka Y. (2012). Quality control of photosystem II: lipid peroxidation accelerates photoinhibition under excessive illumination. *PLoS ONE* 7:e52100 10.1371/journal.pone.0052100 PMC3531424 23300595

[B10] Chow W. S. Thorne S. W. Duniec J. T. Sculley M. J. Boardman N. K. (1980). The stacking of chloroplast thylakoids. Effects of cation screening and binding, studied by the digitonin method. *Arch. Biochem. Biophys.* 201 347–355 10.1016/0003-9861(80)90520-2 7396509

[B11] Demmig-Adams B. (1990). Carotenoids and photoprotection in plants: a role for the xanthophyll zeaxanthin. *Biochim. Biophys. Acta* 1020 1–24 10.1016/0005-2728(90)90088-L

[B12] Duffy C. D. Valkunas L. Ruban A. V. (2013). Light-harvesting processes in the dynamic photosynthetic antenna. *Phys. Chem. Chem. Phys.* 15 18752–18770 10.1039/C3CP51878G 23868502

[B13] Ferjani A. Abe S. Ishikawa Y. Henmi T. Yuka T. Nishi Y. (2001). Characterization of the stromal protease(s) degrading the cross-linked products of the D1 protein generated by photoinhibition of photosystem II. *Biochim. Biophys. Acta* 1503 385–395 10.1016/S0005-2728(00)00233-4 11115650

[B14] Ford R. C. Barber J. (1983). Time-dependent decay and anisotropy of fluorescence from diphenylhexatriene embedded in the chloroplast thylakoid membrane. *Biochim. Biophys. Acta* 722 341–348 10.1016/0005-2728(83)90082-8

[B15] Frank H. A. Bautista J. A. Josue J. S. Young A. J. (2000). Mechanism of nonphotochemical quenching in green plants: energies of the lowest excited singlet states of violaxanthin and zeaxanthin. *Biochemistry* 39 2831–2837 10.1021/bi9924664 10715102

[B16] Franova M. Repakova J. Capkova P. Holopainen J. M. Vattulainen I. (2010). Effects of DPH on DPPC-cholesterol membranes with varying concentrations of cholesterol: from local perturbations to limitations in fluorescence anisotropy experiments. *J. Phys. Chem. B* 114 2704–2711 10.1021/jp908533x 20136066

[B17] Fristedt R. Willig A. Granath P. Crevecoeur M. Rochaix J. D. Vener A. V. (2009). Phosphorylation of photosystem II controls functional macroscopic folding of photosynthetic membranes in *Arabidopsis*. *Plant Cell* 21 3950–3964 10.1105/tpc.109.069435 20028840 PMC2814517

[B18] Genty B. Briantais J.-M. Baker N. R. (1989). The relationship between the quantum yield of photosynthetic electron transport and quenching of chlorophyll fluorescence. *Biochim. Biophys. Acta* 990 87–92 10.1016/S0304-4165(89)80016-9

[B19] Goral T. K. Johnson M. P. Brain A. P. Kirchhoff H. Ruban A. V. Mullineaux C. W. (2010). Visualizing the mobility and distribution of chlorophyll proteins in higher plant thylakoid membranes: effects of photoinhibition and protein phosphorylation. *Plant J.* 62 948–959 10.1111/j.0960-7412.2010.04207.x. 20230505

[B20] Goral T. K. Johnson M. P. Duffy C. D. Brain A. P. Ruban A. V. Mullineaux C. W. (2011). Light-harvesting antenna composition controls the macrostructure and dynamics of thylakoid membranes in *Arabidopsis*. *Plant J.* 69 289–301 10.1111/j.1365-313X.2011.04790.x 21919982

[B21] Gruszecki W. I. Grudzinski W. Gospodarek M. Patyra M. Maksymiec W. (2006). Xanthophyll-induced aggregation of LHCII as a switch between light-harvesting and energy dissipation systems. *Biochim. Biophys. Acta* 1757 1504–1511 10.1016/j.bbabio.2006.08.002 16978579

[B22] Haferkamp S. Haase W. Pascal A. A. Van Amerongen H. Kirchhoff H. (2010). Efficient light harvesting by photosystem II requires an optimized protein packing density in Grana thylakoids. *J. Biol. Chem.* 285 17020–17028 10.1074/jbc.M109.077750 20360011 PMC2878060

[B23] Herbstova M. Tietz S. Kinzel C. Turkina M. V. Kirchhoff H. (2012). Architectural switch in plant photosynthetic membranes induced by light stress. *Proc. Natl. Acad. Sci. U.S.A.* 109 20130–20135 10.1073/pnas.1214265109 23169624 PMC3523818

[B24] Horton P. Ruban A. V. Rees D. Pascal A. A. Noctor G. Young A. J. (1991). Control of the light-harvesting function of chloroplast membranes by aggregation of the LHCII chlorophyll-protein complex. *FEBS Lett.* 292 1–4 10.1016/0014-5793(91)80819-O 1959588

[B25] Horton P. Ruban A. V. Walters R. G. (1996). Regulation of light harvesting in green plants. *Annu. Rev. Plant Physiol. Plant Mol. Biol.* 47 655–684 10.1146/annurev.arplant.47.1.655 15012304

[B26] Ishikawa Y. Nakatani E. Henmi T. Ferjani A. Harada Y. Tamura N. (1999). Turnover of the aggregates and cross-linked products of the D1 protein generated by acceptor-side photoinhibition of photosystem II. *Biochim. Biophys. Acta* 1413 147–158 10.1016/S0005-2728(99)00093-6 10556627

[B27] Johnson M. P. Goral T. K. Duffy C. D. P. Brain A. P. R. Mullineaux C. W. Ruban A. V. (2011). Photoprotective energy dissipation involves the reorganization of photosystem II light-harvesting complexes in the grana membranes of spinach chloroplasts. *Plant Cell* 23 1468–1479 10.1105/tpc.110.081646 21498680 PMC3101555

[B28] Kamata T. Hiramoto H. Morita N. Shen J. R. Mann N. H. Yamamoto Y. (2005). Quality control of Photosystem II: an FtsH protease plays an essential role in the turnover of the reaction center D1 protein in *Synechocystis* PCC 6803 under heat stress as well as light stress conditions. *Photochem. Photobiol. Sci.* 4 983–990 10.1039/b506068k 16307111

[B29] Khatoon M. Inagawa K. Pospisil P. Yamashita A. Yoshioka M. Lundin B. (2009). Quality control of photosystem II: thylakoid unstacking is necessary to avoid further damage to the D1 protein and to facilitate D1 degradation under light stress in spinach thylakoids. *J. Biol. Chem.* 284 25343–25352 10.1074/jbc.M109.007740 19617353 PMC2757236

[B30] Kirchhoff H. Haferkamp S. Allen J. F. Epstein D. B. Mullineaux C. W. (2008a). Protein diffusion and macromolecular crowding in thylakoid membranes. *Plant Physiol.* 146 1571–1578 10.1104/pp.107.115170 18287489 PMC2287334

[B31] Kirchhoff H. Lenhert S. Buchel C. Chi L. Nield J. (2008b). Probing the organization of photosystem II in photosynthetic membranes by atomic force microscopy. *Biochemistry* 47 431–440 10.1021/bi7017877 18067327

[B32] Kirchhoff H. Sharpe R. M. Herbstova M. Yarbrough R. Edwards G. E. (2013). Differential mobility of pigment-protein complexes in granal and agranal thylakoid membranes of C(3) and C(4) plants. *Plant Physiol.* 161 497–507 10.1104/pp.112.207548 23148078 PMC3532279

[B33] Komayama K. Khatoon M. Takenaka D. Horie J. Yamashita A. Yoshioka M. (2007). Quality control of Photosystem II: cleavage and aggregation of heat-damaged D1 protein in spinach thylakoids. *Biochim. Biophys. Acta* 1767 838–846 10.1016/j.bbabio.2007.05.001 17543883

[B34] Komenda J. Barker M. Kuvikova S. De Vries R. Mullineaux C. W. Tichy M. (2006). The FtsH protease slr0228 is important for quality control of photosystem II in the thylakoid membrane of *Synechocystis* sp. PCC 6803. *J. Biol. Chem.* 281 1145–1151 10.1074/jbc.M503852200 16286465

[B35] Kota Z. Horvath L. I. Droppa M. Horvath G. Farkas T. Pali T. (2002). Protein assembly and heat stability in developing thylakoid membranes during greening. *Proc. Natl. Acad. Sci. U.S.A.* 99 12149–12154 10.1073/pnas.192463899 12213965 PMC129413

[B36] Lentz B. R. (1989). Membrane fluidity as detected by diphenylhexatriene probes. *Chem. Phys. Lipids* 50 171–190 10.1016/0009-3084(89)90049-2

[B37] Li X. P. Bjorkman O. Shih C. Grossman A. R. Rosenquist M. Jansson S. (2000). A pigment-binding protein essential for regulation of photosynthetic light harvesting. *Nature* 403 391–395 10.1038/35000131 10667783

[B38] Lupinkova L. Komenda J. (2004). Oxidative modifications of the Photosystem II D1 protein by reactive oxygen species: from isolated protein to cyanobacterial cells. *Photochem. Photobiol.* 79 152–162 10.1562/0031-8655(2004)079<0152:OMOTPI>2.0.CO;2 15068028

[B39] Mori H. Yamamoto Y. (1992). Deletion of antenna chlorophyll-a-binding proteins Cp43 and Cp47 by tris-treatment of Ps-Ii membranes in weak light – evidence for a photo-degradative effect on the Ps-Ii components other than the reaction center-binding proteins. *Biochim. Biophys. Acta* 1100 293–298 10.1016/0167-4838(92)90484-U

[B40] Mori H. Yamashita Y. Akasaka T. Yamamoto Y. (1995). Further characterization of the loss of antenna chlorophyll-binding protein Cp43 from photosystem-Ii during donor-side photoinhibition. *Biochim. Biophys. Acta * 1228 37–42 10.1016/0005-2728(94)00156-Y

[B41] Ohira S. Morita N. Suh H. J. Jung J. Yamamoto Y. (2005). Quality control of photosystem II under light stress – turnover of aggregates of the D1 protein in vivo. *Photosynth. Res.* 84 29–33 10.1007/s11120-004-7310-7 16049751

[B42] Ruban A. V. Calkoen F. Kwa S. L. S. Vangrondelle R. Horton P. Dekker J. P. (1997). Characterisation of LHC II in the aggregated state by linear and circular dichroism spectroscopy. *Biochim. Biophys. Acta* 1321 61–70 10.1016/S0005-2728(97)00047-9

[B43] Ruban A. V. Horton P. (1992). Mechanism of delta-Ph-dependent dissipation of absorbed excitation-energy by photosynthetic membranes. 1. Spectroscopic analysis of isolated light-harvesting complexes.* Biochim. Biophys. Acta* 1102 30–38 10.1016/0005-2728(92)90061-6

[B44] Sarcina M. Bouzovitis N. Mullineaux C. W. (2006). Mobilization of photosystem II induced by intense red light in the *Cyanobacterium Synechococcus* sp. PCC7942. *Plant Cell* 18 457–464 10.1105/tpc.105.035808 PMC1356551 16387835

[B45] Sarcina M. Murata N. Tobin M. J. Mullineaux C. W. (2003). Lipid diffusion in the thylakoid membranes of the *Cyanobacterium Synechococcus* sp.: effect of fatty acid desaturation. *FEBS Lett.* 553 295–298 10.1016/S0014-5793(03)01031-7 14572639

[B46] Stoitchkova K. Busheva M. Apostolova E. Andreeva A. (2006). Changes in the energy distribution in mutant thylakoid membranes of pea with modified pigment content. II. Changes due to magnesium ions concentration.* J. Photochem. Photobiol. B* 83 11–20 10.1016/j.jphotobiol.2005.11.011 16406551

[B47] Taiz L. Zeiger E. (2006). *Plant Physiology*. Sunderland, MA: Sinauer Associates, Inc

[B48] Tardy F. Havaux M. (1997). Thylakoid membrane fluidity and thermostability during the operation of the xanthophyll cycle in higher-plant chloroplasts. *Biochim. Biophys. Acta* 1330 179–193 10.1016/S0005-2736(97)00168-5 9408171

[B49] Vener A. V. (2007). Environmentally modulated phosphorylation and dynamics of proteins in photosynthetic membranes. *Biochim. Biophys. Acta* 1767 449–457 10.1016/j.bbabio.2006.11.007 17184728

[B50] Wilk L. Grunwald M. Liao P. N. Walla P. J. Kuhlbrandt W. (2013). Direct interaction of the major light-harvesting complex II and PsbS in nonphotochemical quenching. *Proc. Natl. Acad. Sci. U.S.A.* 110 5452–5456 10.1073/pnas.1205561110 23509270 PMC3619350

[B51] Yamamoto H. Y. Kamite L. (1972). The effects of dithiothreitol on violaxanthin de-epoxidation and absorbance changes in the 500-nm region. *Biochim. Biophys. Acta* 267 538–543 10.1016/0005-2728(72)90182-X 5047136

[B52] Yamamoto Y. (2001). Quality control of photosystem II. *Plant Cell Physiol.* 42 121–128 10.1093/pcp/pce022 11230565

[B53] Yamamoto Y. Akasaka T. (1995). Degradation of antenna chlorophyll-binding protein CP43 during photoinhibition of photosystem II. *Biochemistry* 34 9038–9045 10.1021/bi00028a012 7619803

[B54] Yamamoto Y. Aminaka R. Yoshioka M. Khatoon M. Komayama K. Takenaka D. (2008). Quality control of photosystem II: impact of light and heat stresses. *Photosynth. Res.* 98 589–608 10.1007/s11120-008-9372-4 18937045

[B55] Yamamoto Y. Ford R. C. Barber J. (1981). Relationship between thylakoid membrane fluidity and the functioning of pea chloroplasts : effect of cholesteryl hemisuccinate. *Plant Physiol.* 67 1069–1072 10.1104/pp.67.6.1069 16661811 PMC425836

[B56] Yamamoto Y. Nishi Y. Yamasaki H. Uchida S. Ohira S. (2004). Assay of photoinhibition of photosystem II and protease activity. *Methods Mol. Biol.* 274 217–227 10.1385/1-59259-799-8:217 15187282

[B57] Yoshioka M. Nakayama Y. Yoshida M. Ohashi K. Morita N. Kobayashi H. (2010). Quality control of photosystem II: FtsH hexamers are localized near photosystem II at grana for the swift repair of damage. *J. Biol. Chem.* 285 41972–41981 10.1074/jbc.M110.117432 20921219 PMC3009923

[B58] Yoshioka M. Uchida S. Mori H. Komayama K. Ohira S. Morita N. (2006). Quality control of photosystem II – cleavage of reaction center D1 protein in spinach thylakoids by FtsH protease under moderate heat stress. *J. Biol. Chem.* 281 21660–21669 10.1074/jbc.M602896200 16735503

